# Differentiating naturally occurring and disease associated autoantibodies in MPO-ANCA vasculitis through immunogenomic and epitope comparisons

**DOI:** 10.3389/fmed.2026.1784607

**Published:** 2026-06-02

**Authors:** Dhruti P. Chen, Young-Hyun Moon, Bridget A. Peck, Justin J. Taylor, Ismael Gomez-Martinez, Mark G. Woodcock, Benjamin G. Vincent, Ronald J. Falk, John S. Poulton, Donna O. Bunch

**Affiliations:** 1 University of North Carolina-Chapel Hill, School of Medicine, Chapel Hill, NC, United States; 2 UNC SOM Department of Medicine, UNC Kidney Center, Chapel Hill, NC, United States; 3 Department of Medicine, University of Virginia, Charlottesville, VA, United States; 4 Division of Infectious Disease and International Health, Department of Medicine, University of Virginia, Charlottesville, VA, United States; 5 UNC SOM Bioinformatics and Analytics Research Collaborative, Chapel Hill, NC, United States; 6 UNC Lineberger Comprehensive Cancer Center, Chapel Hill, NC, United States; 7 UNC Department of Microbiology and Immunology, Chapel Hill, NC, United States

**Keywords:** ANCA vasculitis, autoantibodies, epitope, MPO, PR3, B cells, tetramer

## Abstract

**Introduction:**

ANCAs are diagnostic and pathogenic in ANCA-associated vasculitis however ANCAs can persist or reappear in clinical remission. It is a known phenomenon that naturally occurring autoantibodies, including ANCAs, are also detectable in healthy individuals. That autoantibodies do not directly correlate with clinical disease suggests that characteristics of the autoantibody response, rather than its mere presence, may determine pathogenicity. We aimed to define immunogenomic and epitope-level differences between naturally occurring and disease-associated MPO-ANCA.

**Methods:**

Patients with MPO-ANCA vasculitis (active disease and long-term remission off therapy) and healthy individuals were enrolled at a single center. Peripheral blood mononuclear cells were screened using highly sensitive methods to isolate MPO specific B cells from healthy individuals and patients with MPO-ANCA. Sorted cells were expanded in vitro with antigen stimulation, and supernatants were tested for MPO-ANCA IgG. Bulk B cell receptor (BCR) sequencing of heavy and light chains was performed on autoantigen-specific and control B cells. Serum IgG from the same cohort was used to map MPO epitopes using a yeast surface display library of overlapping MPO-derived peptides.

**Results:**

We found that autoantigen-specific B cells were present at similar frequencies in patients with active disease, patients in long-term remission off therapy, and healthy individuals. However, only MPO specific B cells from MPO-ANCA patients produced high levels of MPO-ANCA in vitro, whereas MPO specific B cells from healthy individuals did not. MPO specific B cells from patients exhibited shared immunogenomic features such as reduced clonotype diversity and clonal dominance compared with non-MPO B cells, and there was greater overlap in CDR3 sequences. Epitope mapping revealed that healthy individuals had naturally occurring MPO-reactive IgG, however patient IgG conferred distinct MPO epitope signatures.

**Discussion:**

Our findings demonstrate that naturally occurring and disease-associated MPO-ANCA differ in both BCR repertoire features and epitope specificity. These data support the concept that immunological remission in MPO-ANCA vasculitis cannot be defined solely by the presence or absence of ANCAs, but depends on qualitative differences in the autoantibody response, including BCR sequence and epitope targeting.

## Introduction

In Anti-Neutrophil Cytoplasmic Antibody (ANCA) vasculitis, autoantigen-specific B cells make and secrete measurable IgG autoantibodies targeting one of two autoantigens: proteinase 3 (PR3) and myeloperoxidase (MPO) (1). The autoantibodies are markers for diagnostic purposes and a key part of ANCA immunopathogenesis. ANCAs have been shown to be pathogenic in *in vitro* studies, animal models (2) and case reports of placental transfer (3). In patients who achieve remission and therapy is stopped, ANCAs often reappear at measurable levels without clinical disease and multiple studies have shown that levels do not directly correlate with disease activity (4–8). Lack of clinical disease despite circulating autoantibodies suggest potential alterations in some characteristic(s) of the autoantibody that mitigate pathogenesis and/or the presence of protective factors during remission. Notably, there is a range of autoantibody levels above which ANCAs are considered clinically significant to reflect the finding that healthy individuals also produce ANCAs (9). Thus far, clinical testing measures ANCA IgG levels, and that will be the focus of this paper. The presence of naturally occurring autoantibodies in healthy individuals as well as lack of disease in the presence of ANCAs in some remission patients raises the question of what autoantibody characteristics elicit a pathologic response (10). The prevailing paradigm that defines immunological remission solely by the absence of IgG autoantibodies should be expanded to address a critical distinction between naturally occurring autoantibodies and those associated with disease. Naturally occurring autoantibodies have fascinated researchers and there is much literature surrounding hypotheses about their role in healthy individuals. One suggested physiological role is clearance of apoptotic cells or debris by binding and enhancing phagocytic uptake to help prevent release of pro-inflammatory intracellular contents and maintain homeostasis (11). This may further regulate and modulate immune activity by influencing signaling pathways and suppressing excessive inflammation or even through shaping the B cell repertoire (12, 13). We focus our attention on differentiating these (ANCA) autoantibodies from those found in people with known disease.

We propose that naturally occurring and disease-associated ANCA differ in the protein sequences of the autoantibodies themselves. Autoantibodies are initially present as membrane-bound B cell receptors (BCRs) and consist of a heavy and light chain with constant and variable regions. Within the variable regions, there are complementarity determining regions 1–3 (CDR1-3) that contact the antigen and are the most diversified regions. Specifically, CDR3 is the major determinant of antigen binding specificity and in autoimmunity has been found to have unique sequence length changes and physiological properties (14). We hypothesize that the BCR sequences differentiate naturally occurring autoantibodies (from healthy individuals) and disease-associated antibodies.

In prior ANCA vasculitis studies, BCR sequencing of the total B cell populations (from patients) did not yield skewed IGHV gene usage, B cell clonal expansion, or CDR3 length differences compared to healthy individuals. Another study using B cell subsets identified clinically significant differences in BCR repertoire in SLE samples but not in samples from patients with ANCA vasculitis (15, 16). We propose that rather than total B cells or similarly large subsets, BCR sequencing of only autoantigen-specific B cells would more accurately describe the ANCA immunogenomic signature for better comparison of disease-related autoantibodies to naturally occurring autoantibodies. Isolation of pure populations of antigen-specific B cells is critical for accurate studies of ANCA BCR diversity but difficult to accomplish. We therefore developed a precise method to identify and isolate rare circulating autoantigen specific B cells to characterize their BCRs sequences.

Previous studies of autoimmune diseases, including ANCA vasculitis, suggest an association between disease activity and autoantibody epitope-specificity. BCR sequences encode the antibody that binds an autoantigen epitope. Therefore, a corollary hypothesis is that ANCA epitope specificity varies among naturally occurring and disease-associated ANCA. We tested this hypothesis using serum IgG on a custom yeast surface display platform to determine MPO-specific epitopes. Together, these hypotheses investigate the fundamental differences in the nature of the autoantibodies themselves with detailed characterizations of the autoantibodies and the B cells that generate them. Our findings support the view that immunological remission cannot be defined by presence or absence of autoantibodies alone but is likely achieved by a combination of factors which include alterations in the autoantibody sequences and their epitope binding characteristics.

## Methods

### Patients and healthy controls

Patients with ANCA vasculitis were enrolled at the University of North Carolina-Chapel Hill (UNCCH) clinics and followed in the Glomerular Disease Collaborative Network. Patients were diagnosed according to Chapel Hill Consensus Conference criteria (17, 18). Subjects were recruited, provided informed, written consent, and participated according to UNCCH Office of Human Research Ethics Institutional Review Board guidelines (study no. 97–0523). Disease activity and remission were confirmed through chart review as previously published by our group. Disease activity was defined by the 2003 Birmingham Vasculitis Activity Score (BVAS) and clinical activity (19). Patients with a BVAS of 0 and no clinical or laboratory evidence of active disease within 3 months were considered in remission. Active disease was defined as BVAS >0 with clinical and/or laboratory evidence of disease. Complete remission after initial diagnosis was required prior to relapse. ANCA serotypes were assessed by indirect immunofluorescence (IF) with positive cytoplasmic (C-ANCA) or perinuclear (P-ANCA) staining and antigen-specific PR3 and MPO enzyme-linked immunosorbent assays (ELISA) (20). MPO- and/or P-ANCA were classified together and PR3- and/or C-ANCA were classified together. Patients with only P-ANCA positivity prior to MPO availability were required to have negative ANA. Long-term remission off therapy (LTROT) was defined as previously reported (21). LTROT patients had no active disease 3 months prior to or following the sample date and no immunosuppressive therapy 6 months prior to sample date. LTROT patients who previously received rituximab had to be off therapy at least 1 year prior to sample or demonstrate evidence of CD19+ B cell return. Patients who had GFR < 20 or considered end stage renal disease were excluded. Healthy individuals were consented prior to blood donation. Limited clinical data are available in Supplementary Table 1. Samples for healthy individuals were obtained with consent from community dwelling adults who donate blood products at our blood bank. These donors are screened for routine vitals and transfusion transmitted infections. Additionally, donors with cancers such as lymphoma or leukemia are excluded. We attempted to age match the cohorts (average age 52 years and 48 years in patients and healthy individuals respectively).

### Cell preparation and cell surface staining for identification of autoantigen-specific B cells

PBMCs were purified from heparinized peripheral blood samples by centrifugation in cell preparation tubes (Becton Dickinson and Company, Franklin Lakes, NJ). Cells were washed in PBS, resuspended in Hank’s buffered salt solution (with 2% FCS). For evaluation of autoantigen-specific B cells, cells were stained with CD19 and biotinylated autoantigen followed by detection with fluorescently labeled streptavidin. After fixation with 1% paraformaldehyde, cells were analyzed using a FACSCalibur flow cytometer. Controls including Streptavidin only and “other ANCA antigen” were used to establish positivity for autoantigen binding. Data were analyzed with Summit (DakoCytomation, Carpinteria, CA) or FlowJo (Treestar, Ashland, OR) software.

### Tetramer labeling, FACS, and *in vitro* expansion and ELISA

Cryopreserved PBMCs for immunophenotyping or other experiments: cells were thawed quickly at 37 °C until only one ice crystal remained and resuspended with dropwise addition of cell media (IMDM, 10% FBS, GlutaMAX, beta-mercaptoethanol). After 2 washes in media, cells were allowed to rest overnight at 37 °C with 5% CO2 prior to staining or other experimental procedures. Viability was assessed by trypan blue exclusion. Only thawed PBMCs with greater than 90% viability were used in further analyses. MPO tetramers were developed by Dr. Justin Taylor as previously published (22). The tetramers were validated using beads coated with anti-MPO antibodies (Agilent, polyclonal rabbit anti-human myeloperoxidase, cat number Dako A039) (Supplementary Figure 1). For flow sorting, cells were labeled first with decoy tetramers to identify nonspecific streptavidin or fluorophore binding cells. MPO tetramers were then added to identify autoantigen-specific cells and the population was enriched for fluorophore-binding cells via magnetic bead selection (APC Positive Selection Kit II, Cat 17681). The cells were additionally labeled with CD19 to identify B cells prior to flow sorting using a MACSQuant Tyto cell sorter. The B cells were gated for tetramer positivity and exclusion of the decoy positive population for sorting. Following sorting, cells were cultured with media containing CD40L (Bio-Techne, 6245-CL, 0.5 μg/mL), CpG oligodeoxynucleotide (InvivoGen, tlrl-2006, 1 μg/mL), and MPO antigen (Elastin, No. MY862, 1 ng/mL) in ImmunoCult Human B cell expansion kit media (StemCell Catalog #100-0645). Cells expanded *in vitro* with antigen stimulation were harvested after 14 days and underwent RNA isolation for library preparation and bulk BCR sequencing (Illumina MiSeq). Anti-MPO antibodies were detected in ELISA using native human MPO (Elastin Products Company, Inc., Catalog No. MY862) as described previously (23, 50). Plates were incubated with 100 μL/well of cell culture supernatants. Anti-human IgG (Millipore, Goat anti-Human IgG, alkaline phosphatase conjugated, AP112A) were used as secondary antibodies (1:20,000). Healthy control plasma and media was used as a negative control. Patient-matched plasma served as the positive controls. Reactivity was detected by chemiluminescence assay.

### Yeast epitope mapping

Epitope mapping was performed using our custom yeast surface display library and approach (24). Detailed protocols are available in our previous report; the primary steps are as follows. Sera from healthy individuals or ANCA patients were depleted of non-specific antibodies (anti-yeast antibodies) by incubation overnight at 4C with 10^8^ yeast expressing a FLAG tag; 20 μL serum in 240 μL PBS with 0.1% BSA. 50 μL of the depleted serum was then incubated with 5×10^6^ yeast expressing a custom peptide library. This library is similar to that previously described in the content of the 30amino acid overlapping peptides derived from MPO’s protein sequence (24). However, we improved upon that design by taking advantage of the redundant nature of DNA codons to encode each peptide in the new library using 4 unique DNA sequences. This provides 4 technical replicates within each experiment. Following incubation with patient or healthy serum, the yeast cells were washed then stained with anti-human IgG fluorescent secondary antibody (Invitrogen) to label cells expressing reactive peptides. Those cells were isolated on a Becton Dickinson FACSMelody and expanded for 3 days in culture. The plasmids were extracted and the peptide-coding region was amplified by PCR, followed by NGS and mapping of peptides to MPO, as previously described (24). In this study, we only report the signal for peptides that had positive values for all four DNA-based variants of each peptide. Heat map and Principal Components Analysis were generated using GraphPad Prism 10.4.2.

### Immunogenomics analysis

Bulk BCR-seq FASTQ files were processed using MiXCR (v4.4.2) (25) and the takara-human-rna-bcr-umi-smartseq built-in preset with default settings (26). Shannon diversity was calculated using base R functions. The Immunarch package (0.9.1) (27) was used to quantify the repertoire space occupied by clones from various indices (1:10, 10:100, 101:1000, 1001:3000, 3001–10000). Repertoires were subsequently subsetted according to their chain type (IGH, IGK, IGL) and the occupied repertoire space was calculated as previously described. Repertoire overlap was calculated using the Morisita-Horn Index via the Immunarch package.

## Results

### Autoantigen-specific B cells are present in patients with active disease, disease remission, and healthy individuals

**Figure 1:**
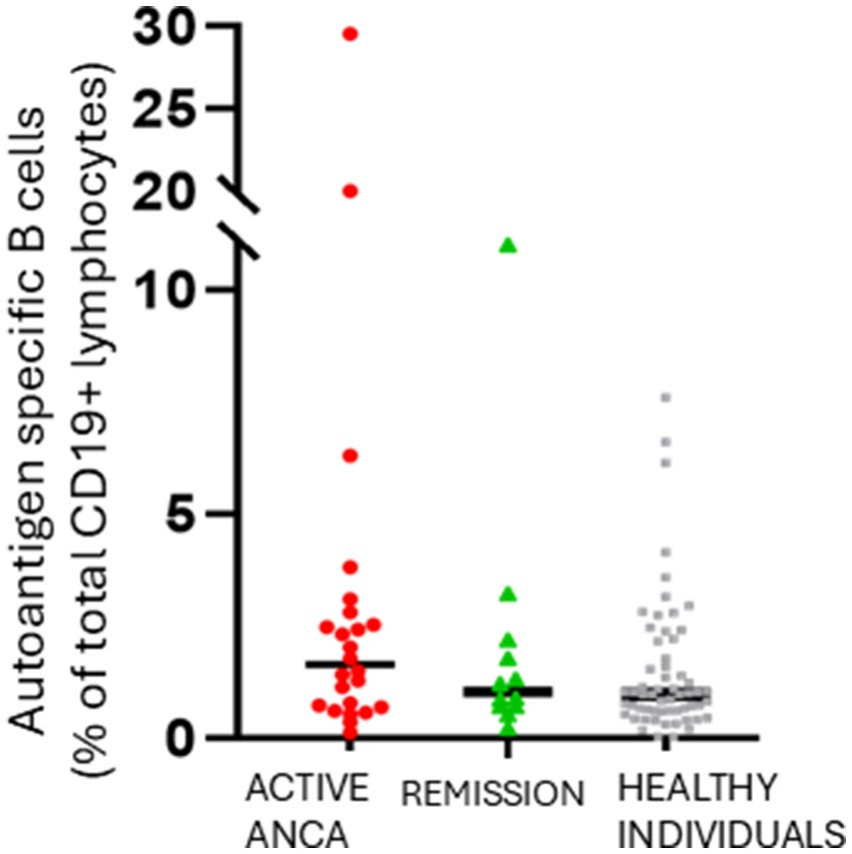
ANCA autoantibodies or autoantigen-specific B cells are identified in ANCA patients with active disease or during remission and in healthy individuals using biotinylated autoantigens (MPO and PR3).

**Figure 2:**
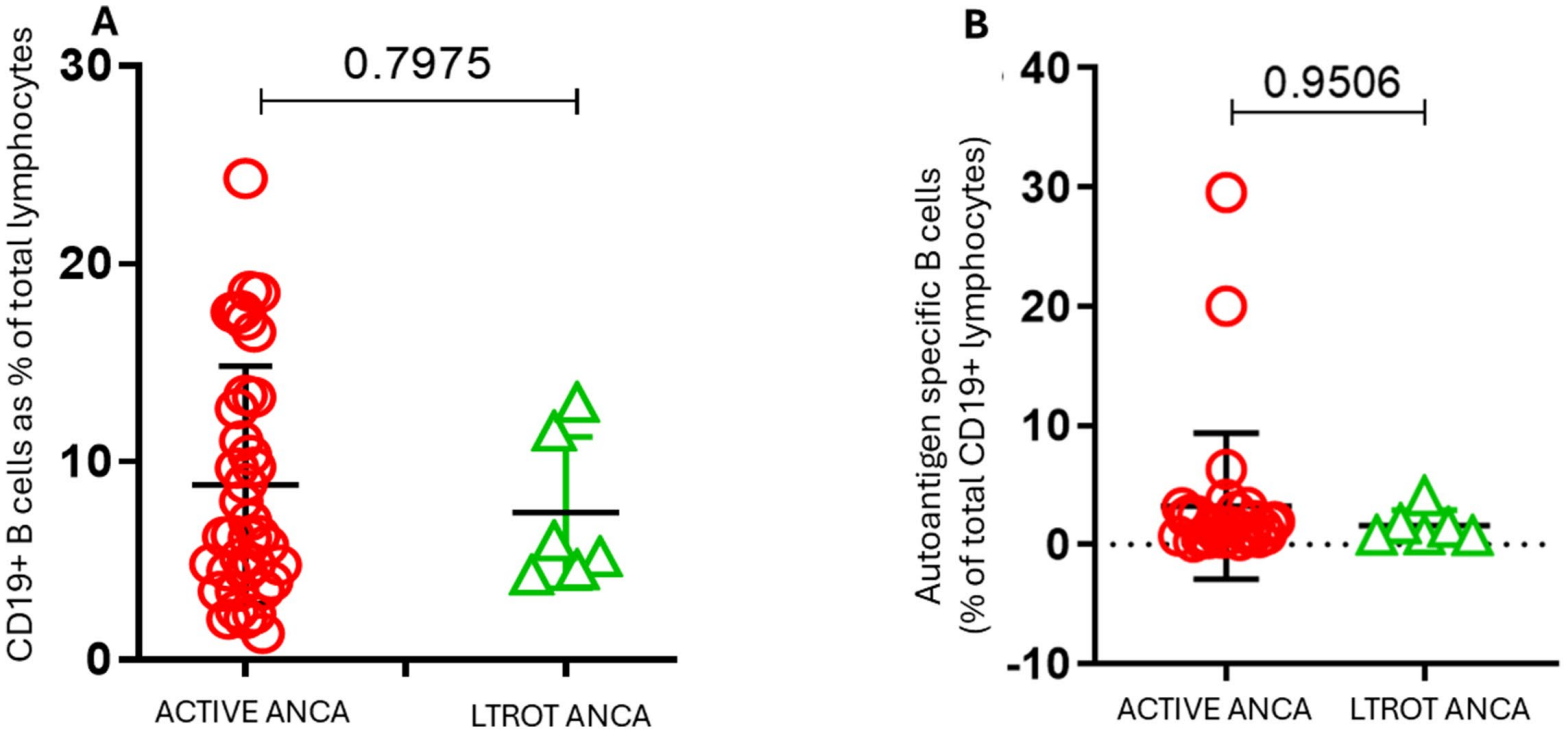
B cells repopulate in patients in remission off therapy to similar levels as patients with active disease. **(A)** Flow cytometry analysis of total B cells (CD19+ lymphocytes) in clinically active ANCA patients (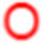) and ANCA patients in long term remission off therapy (LTROT) (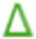) indicates comparables levels of total B cells. **(B)** Autoantigen specific B cells are also present at similar levels in active (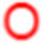) and LTROT patients (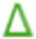), as a percentage of total B cells. [Mann Whitney test (*n* = 21)].

To test the hypothesis that presence of autoantibodies reflects autoantigen-specific B cells, we measured the levels of autoantigen-specific (PR3 or MPO) B cells in samples from patients and healthy individuals and found that all groups have autoantigen-specific B cells present at similar levels (Figure 1). To remove any effect of therapy which could impact B cells, we did a subset analysis to compare patients with active disease and patients in long-term remission off therapy (LTROT), as previously defined. We found that patients in both categories had comparable levels of circulating B cells (Figure 2A) and that ANCA autoantigen-specific B cells were also present during LTROT at similar levels as active patients (Figure 2B). These data support the contention that disease activity is not solely predicated on the presence or absence of autoantigen-specific B cells.

### Isolated autoantigen-specific B cells from patients produce MPO-ANCAs

To refine our ability to sort and study this rare cell type, we developed a precise method using a stable tetramerized antigen probe for high binding avidity and identification of MPO-specific B cells. Previous studies have utilized biotinylated antigens (28), but advancement using tetramers improved specificity for fluorescent activated cell sorting (FACS) and downstream applications such as cell culture and sequencing. Dr. Taylor’s lab constructed fluorescently tagged tetramers (on streptavidin backbone) loaded with whole MPO antigen using a well-established protocol (22). We also simultaneously developed backbone-only tetramers tagged with a different fluorophore to use as “decoys” to engage B cells which bind non-specifically to the non-antigen tetramer components. This method allows for high-resolution detection of MPO-specific B cells while excluding cells binding decoy controls. We used the whole MPO protein to avoid excluding any surface-accessible epitopes.

**Figure 3:**
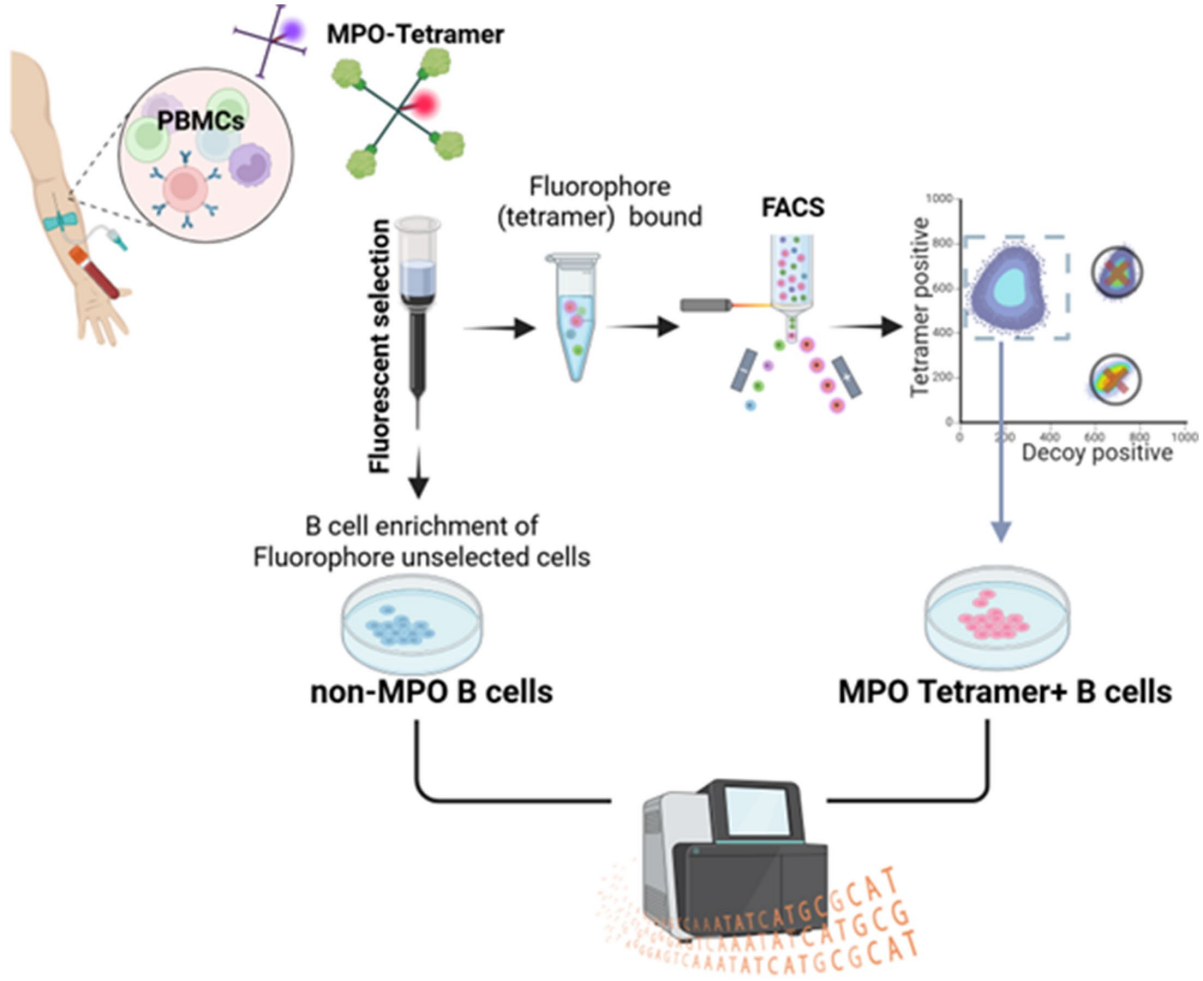
Experimental schematic for the isolation and BCR sequencing of autoantigen specific B cells. Peripheral blood mononuclear cells (PBMCs) from patients or healthy individuals are incubated with fluorescently labeled tetramers. MPO-tetramers are loaded with MPO autoantigen and decoy tetramers with only the streptavidin backbone; the two tetramer forms are tagged with different fluorophores. Tetramer positive cells were separated via fluorophore selection kit prior to sorting via fluorescence-activated cell sorting (FACS) to isolate MPO-tetramer positive cells (excluding decoy or non-specific bound cells). The remaining fluorophore unselected cells underwent B cell enrichment to yield non-MPO B cells as a control population. Both populations underwent in vitro expansion with autoantigen to isolate adequate cells for bulk BCR sequencing Created by Biorender.

**Figure 4:**
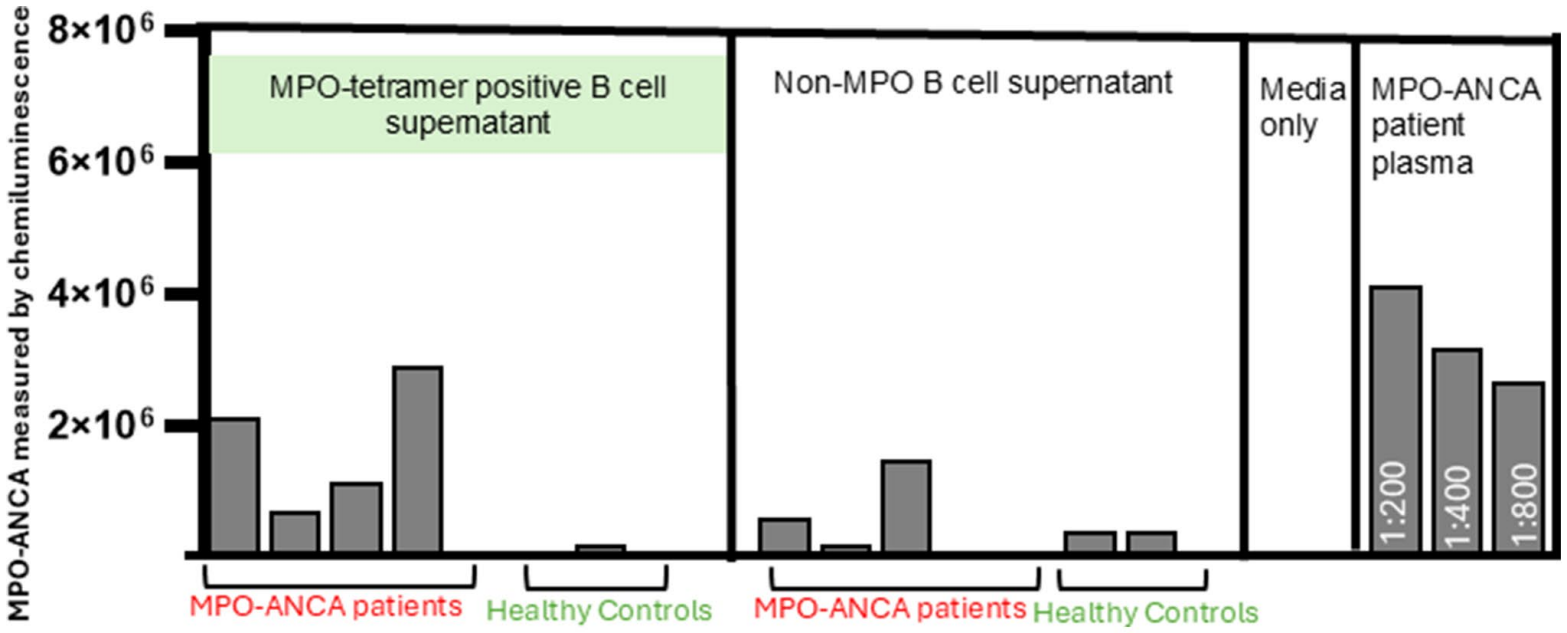
Autoantigen specific B cells from patients but not healthy individuals secrete MPO-ANCAs *in vitro*. Supernatants from expanded autoantigen specific (MPO-tetramer positive) and non-MPO B cells were tested for the presence of anti-MPO IgG by ELISA. Patient derived autoantigen specific B cells (MPO-tetramer positive) produce MPO-ANCAs but autoantigen specific B cells from healthy individuals do not. The non-MPO B cells (tetramer negative) secrete lower levels of MPO-ANCAs, with no clear differences between patients and healthy individuals. As a positive control and comparator, we used diluted plasma from a patient with clinical high titer of MPO-ANCA; dilution indicated on the bar.

We labeled and sorted PBMCs from patients (*n* = 4) with MPO-ANCA vasculitis (active and LTROT) and healthy individuals (*n* = 2) using our MPO-tetramers (Supplementary Table 1). Cells that bound MPO-tetramers were designated as MPO tetramer-positive cells and subsequently compared to two other cell populations from the same individuals, which served as experimental controls: MPO-tetramer-negative cells (decoy tetramer-positive) and other non-MPO B cells (‘non-MPO B cells” were derived by B cell enrichment of cells that did not bind either tetramers or decoys), as shown in the Figure 3 experiment schematic. Sorted cell populations were cultured for 10–14 days with MPO for expansion prior to RNA isolation for bulk BCR sequencing. To validate that the tetramer-positive cells were autoantigen-specific B cells, we assessed the *in vitro* production of MPO ANCA antibodies, similar to previously published studies (29, 30). Supernatants from cultured cells were tested for MPO-ANCAs by ELISA; supernatants from MPO-tetramer positive cells were compared to other B cell supernatants from the same individuals. MPO-tetramer positive cells from patients produced significant levels of MPO-ANCAs, while healthy individuals did not (Figure 4). We used diluted plasma from patients with elevated clinical ANCA titers as positive controls. In contrast to patient-derived MPO-tetramer positive cells, non-MPO B cells did not produce MPO-ANCAs. Non-MPO B cells from the same cohort were used to show that these cells did not produce MPO-ANCAs at levels comparable to the tetramer-positive control. These data suggest that one key difference in the autoantigen-specific B cells from ANCA patients may be their ability to produce greater quantities of ANCA in response to autoantigen stimulation.

### BCR repertoire of the MPO-specific B cell population in patients and healthy individuals

**Figure 5:**
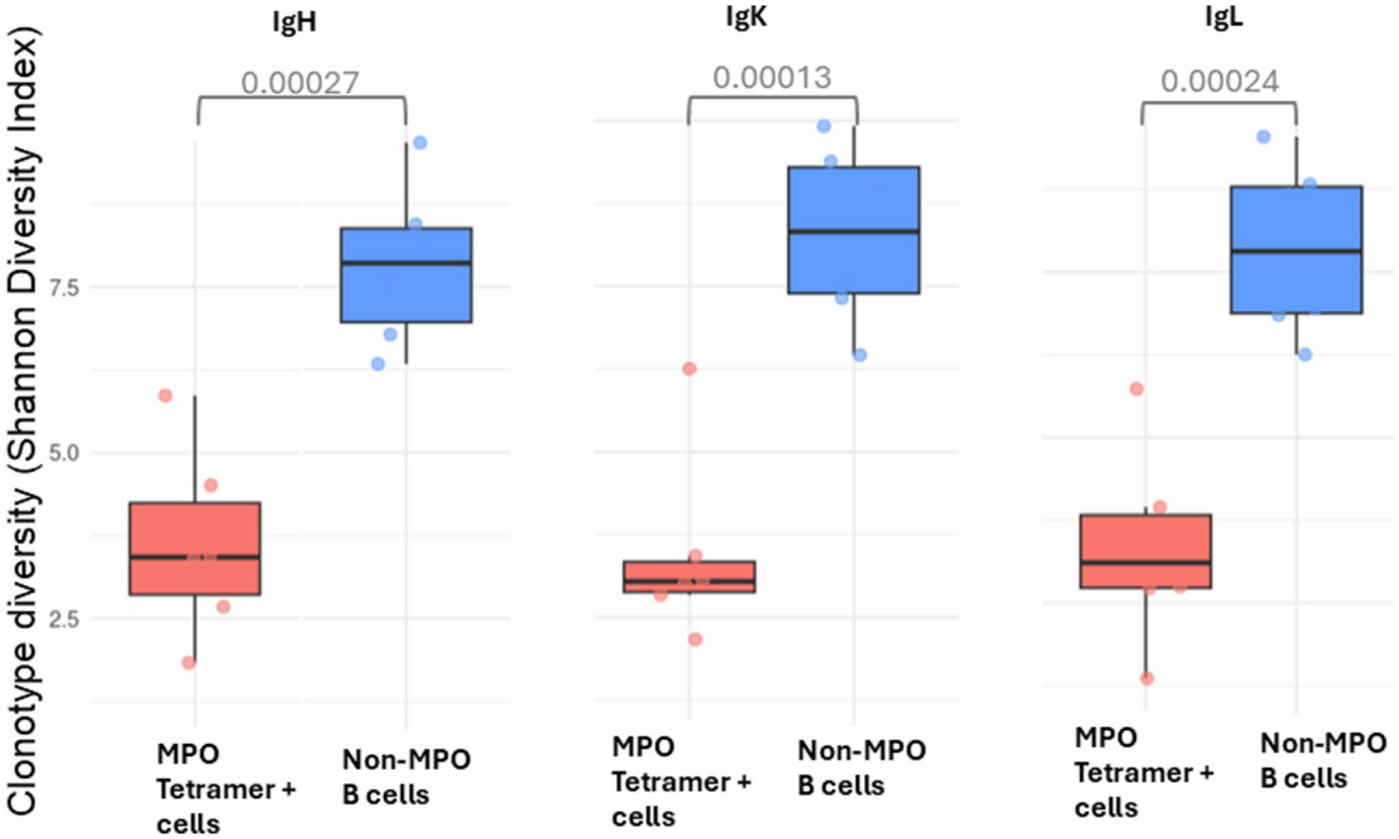
BCRs from autoantigen specific B cells are clonally restricted. Analysis of bulk BCR sequences from MPO specific B cells (tetramer+) and non-MPO B cells indicates significantly less diversity among BCR clonotypes, as measured by Shannon Diversity Index. This difference was detected for all antibody chains (IgH, IgK, IgL).

**Figure 6:**
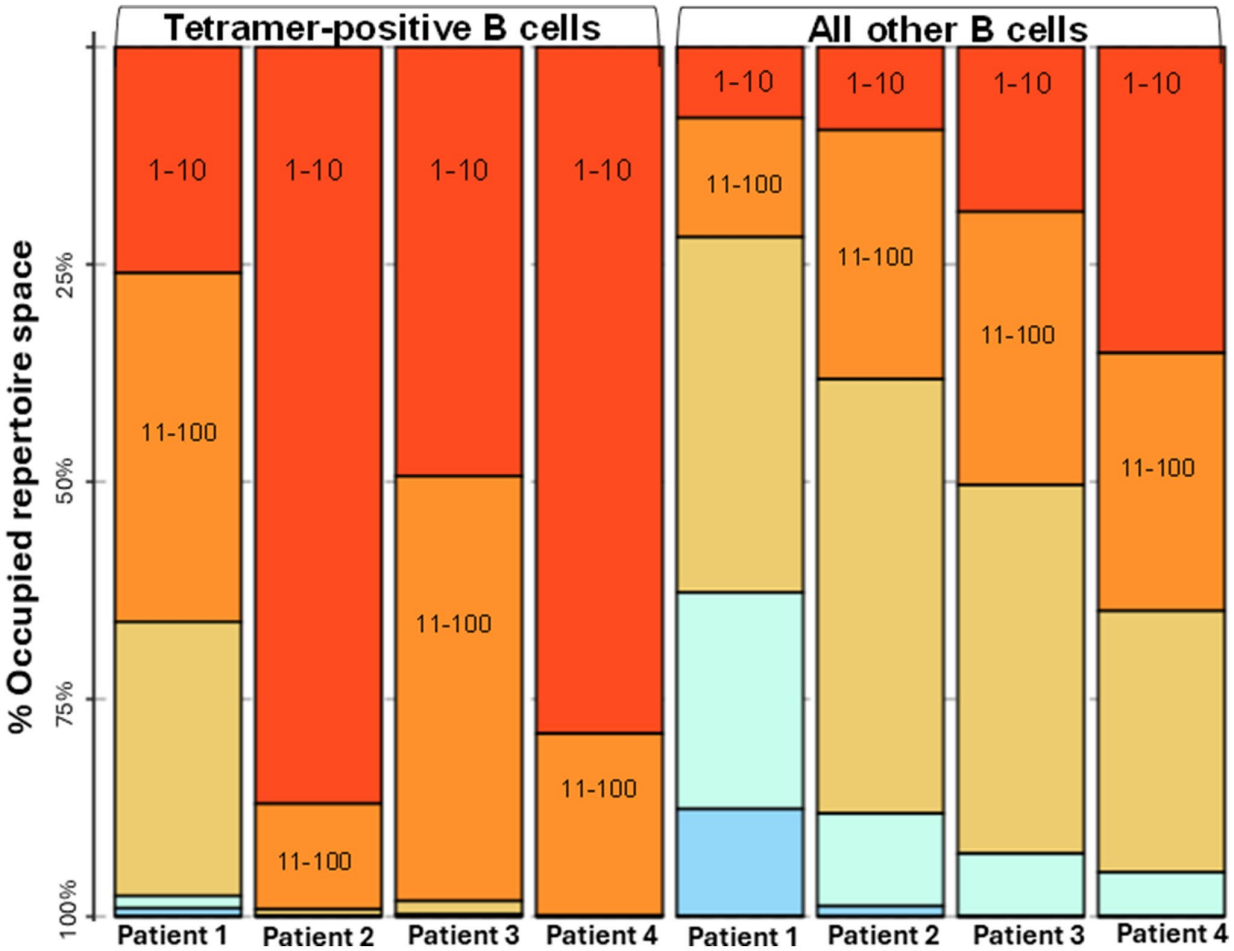
BCRs from autoantigen specific B cells have limited diversity. Stacked bar graphs depict the percent of the BCR sequence repertoire based on the relative abundance of the clones. The left set of graphs are the distributions for the MPO specific B cells (tetramer positive) and the right set are the non-MPO B cells (other) from the same patients. Binning the percent of BCRs sequenced into the top 100 clones (1–10, red 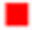 and 11–100, orange 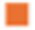 combined), reveals that these clones occupy 60–95% of the total repertoire space in MPO specific B cells; the majority of the BCRs sequenced are represented by only 100 or fewer clones. Conversely, non-MPO (other) B cells have higher clonal diversity, as indicated by the top 100 clones representing a much small proportion of the total BCR repertoire. *n =* 4 MPO-ANCA patients. Tan bin = 101–1,000 clones; light blue 1,001–3,000; dark blue = 3,001–10,000. Created with biorender.com.

**Figure 7:**
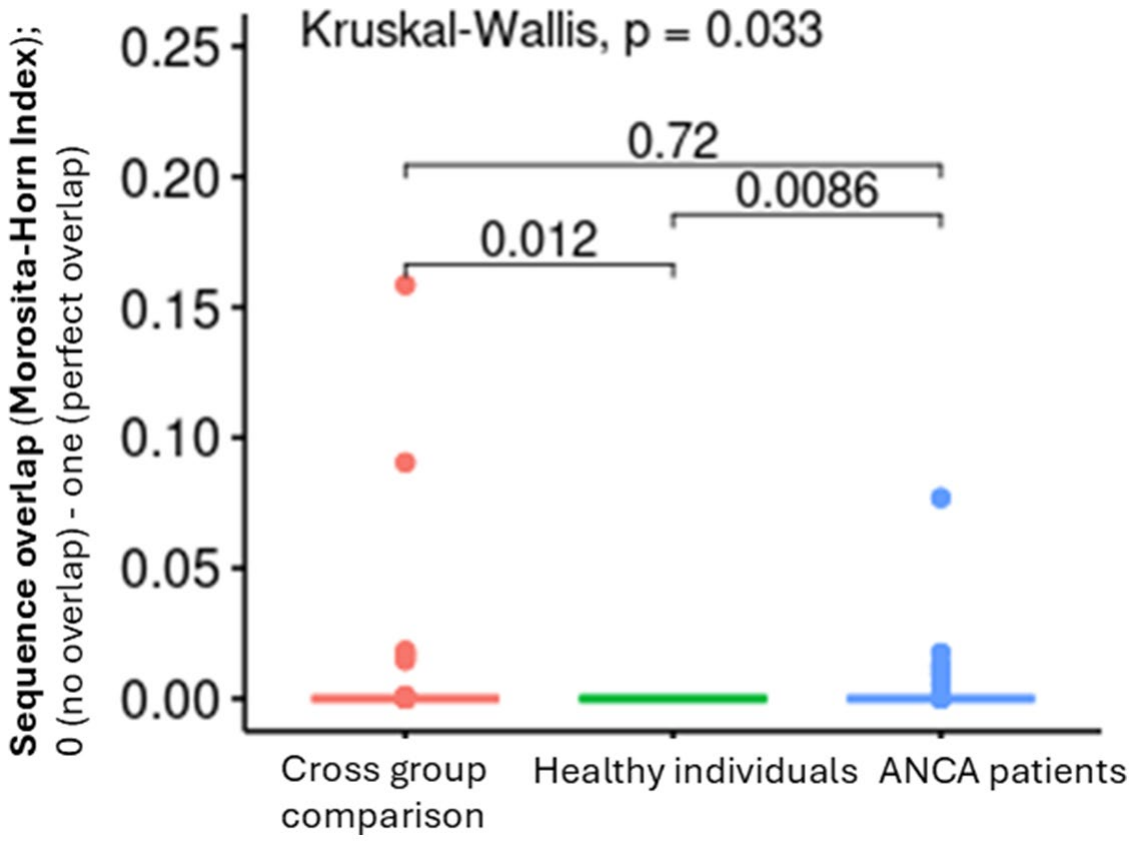
Sequences of complementarity-determining region 3 (CDR3) are more similar among the BCRs of ANCA patients than the BCRs of healthy individuals. Patients had significant overlap (similarity) in CDR3 sequences while there was no overlap among healthy individuals. Morisita-Horn index measures overlap ranging from zero (no overlap) to one (perfect overlap), higher values indicate greater similarities in CDR3 sequences.

We then performed bulk BCR sequencing of heavy and light chains from sorted B cell populations. BCR sequencing from the autoantigen-specific cells showed the BCR repertoire of tetramer-positive cells had skewed clonotype composition compared to control cell populations, as measured through clonal dominance based on a lower Shannon Diversity index (Figure 5). This data is consistent with tetramer-positive cells expanding and reflective of clonal populations of autoantigen-specific B cells. Because we controlled for B cell content, the differences in diversity do not reflect sampling differences. We also found that the top 100 clones occupy much of the repertoire space in tetramer-positive cells from MPO-ANCA patients while the remaining B cells from the patients have greater clonal diversity (Figure 6). The most diverse part of the BCR sequence is CDR3, which plays a crucial role in determining the binding specificity to antigens. We used unique CDR3 amino acid sequences to determine overlap. There were no differences in CDR3 lengths between samples in our study. However, we found that individuals with ANCA vasculitis, but not healthy individuals, had overlap in CDR3, as indicated by higher Morista-Horn overlap index scores (Figure 7). The CDR3 chain length and SD (for heavy and light chains) is included in Supplementary Table 2.

### Signature of MPO epitopes bound by disease-specific autoantibodies vary from epitopes bound by naturally occurring autoantibodies

**Figure 8:**
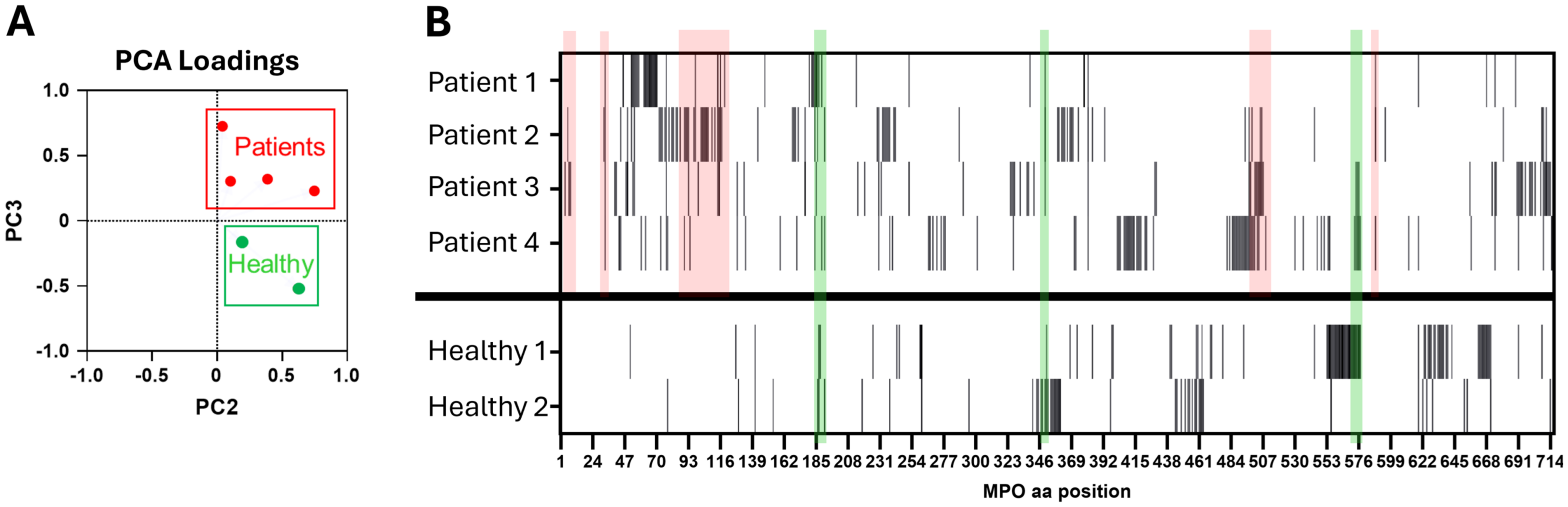
Differences in MPO epitope specificity between ANCA patients and healthy individuals. **(A)** Principal Components Analysis (PCA) of patient and healthy IgG epitopes reveals fundamental differences in the epitope patterns between patients and healthy individuals; patients cluster together and separate from healthy samples along Principal Component (PC) 3. PC1 was examined, but similar to PC2, it did not delineate patient samples from healthy individuals. The proportion of variance in the epitope data explained by PC3 (30%) is on par with that represented by PC1 and PC2 (36% and 34%), indicating PC3 does not represent a minor aspect of our data. **(B)** Heat map of the raw epitope data used in the PCA. Epitope reactivity was determined by IgG binding to a set of 716 overlapping MPO peptides screened via yeast surface display library. Each column represents positive reactivity for the 30 amino acid peptide starting at that position on the MPO protein (*x*-axis). Red highlights are regions of reactivity shared by 2 or more patients but neither healthy individual. Green regions indicate reactivity in one or more patients and healthy controls, representing potential naturally occurring autoantibodies.

A key feature of each BCR is its paratope, which imparts binding specificity to a particular epitope on the autoantigen (31). Given our findings that the BCR sequences from autoantigen-specific B cells had some overlap in ANCA patients but not in healthy controls, we hypothesized that BCR differences between naturally occurring antibodies and disease-associated autoantibodies translated to similar findings in the autoantigenic epitopes. To identify MPO epitopes, we used patient and healthy individual serum samples from the same cohort as above to screen a yeast display library of overlapping peptides derived from the MPO protein sequence (24). The overall IgG epitope signature in healthy individuals is distinct compared to the patient samples, as indicated by Principal Components Analysis (Figure 8A). Examination of epitope locations on MPO identified multiple epitopes bound by IgG from two or more patient samples but not bound by IgG from the healthy individuals (Figure 8B). Notably, IgG from healthy individuals do bind MPO epitopes (Figure 8B), confirming the presence of naturally occurring autoantibodies. This confirms findings from other groups that naturally occurring autoantibodies against MPO are present in healthy individuals (10, 32). Detection of MPO autoantibodies in healthy individuals further reflects the principle that simply measuring autoantibodies is not enough to define disease, as there may be defining epitope characteristics that differ between naturally occurring and disease associated autoantibodies.

## Discussion

We and others have shown that ANCA exist in healthy individuals (9, 10, 32). Here, we show that ANCA-specific autoreactive B cells (which produce the autoantibodies) are also found in healthy individuals. We examine the differences between naturally occurring and disease-associated autoantibodies in MPO-ANCA vasculitis through BCR sequencing and mapping the corresponding epitopes from the same group. In the landscape of ANCA vasculitis, autoantigenic B cell receptors (BCRs) are secreted as autoantibodies targeting one of two autoantigens: PR3 and MPO (1). Some of the existing studies analyzing BCRs from ANCA vasculitis patients have focused on *all* circulating B cells or smaller subsets of the B cell population, but detailed immunogenomic profiling of autoantigen-specific B cells remains limited. One study compared the heavy chain of BCRs (derived from total B cell population) from six different autoimmune diseases including ANCA vasculitis but did not examine autoreactive B cells subsets (15). Another study examined patient derived (*n* = 4) MPO-specific B cells grown in culture and found predominance of MPO IgM-secreting B cells with few MPO IgG secreting cells and no evidence of oligoclonal response (33). A more recent study reported scRNA-Seq data from BCRs of PR3-specific B cells from a single patient yielding 19 PR3+ cells and showed that a majority of the sequences were not clonally related (34). Overall, these studies are limited by small cell numbers from small cohorts. In other diseases, shared BCR clonotypes indicate a common immune response among patients (35). Functional antibodies within individuals show constraint in the V(D)J recombination and immune selection suggesting that certain heavy and light chains are selected for specific antigens (36). In mice with spontaneous crescentic glomerulonephritis, anti-MPO antibody light chains share sequence similarities and influence antigen binding (37). These studies serve as precedent to further understand BCRs in ANCA vasculitis.

Our study expands beyond the current knowledge as we used a highly specific tool to label and sort MPO-specific B cells. We found that rates of autoantigen-specific B cells are similar to previously published studies. Autoantigen-specific B cells during an immune response can represent a low percentage of the total B cell population, making it challenging to isolate and study them (38). This is consistent with reports in ANCA vasculitis (28, 39). We showed there are clonal populations of autoantigen-specific B cells in patients with MPO-ANCA vasculitis. Our current findings suggest that specific antigen binding regions of BCRs from the autoreactive B cells of ANCA patients share sequence similarity. Within the BCRs, there are variable and constant regions. The variable region of each antibody light and heavy chain contains three complementarity-determining regions (CDR1, CDR2, CDR3) which contact the antigen (40). CDR3 is the most highly diversified region and spans the V-D-J recombination junction in the heavy chain and V-J region in the light chain. It is subject to random junctional diversity through mutations and somatic hypermutations. Given the chance of random mutations, conserved or overlapping parts of the CDR3 from autoantigen-specific B cells is an important finding. CDR3 length of the heavy chain has been shown to be increased in some autoimmune diseases but was not observed in ANCA vasculitis (15). While our data shows BCR overlap in patients, there is still variability in BCRs among patients. This is not surprising given the nature of B cell development and somatic hypermutation. Interestingly we found that while there were MPO tetramer-positive cells found in healthy individuals, these cells did not produce high levels of MPO-ANCAs *in vitro*. This may reflect a difference in cell type, though we were limited in our ability to immunophenotype the cells as they were harvested for RNA. Additional limitations of this study stem from insufficient quantities of MPO antibodies harvested during *in vitro* expansion for epitope mapping or functional assays which are needed to parse out the underlying biological differences. Future studies will hopefully be able to address these questions.

Circulating autoantigen-specific B cells are a rare subset within the B cell population, making up maximally 2–7% of total B cells (which are on average 5–10% of total circulating PBMCs). A strength of our study is that we were able to identify and successfully sort a relatively large number of autoantigen-specific B cells for bulk BCR sequencing. While bulk BCR sequencing is advantageous in that more cells are included to cover a larger immune repertoire, there are now technological advances that allow the same approach to be applied to single-cell sequencing to obtain BCR and transcriptomic data concurrently. In healthy individuals, systems immunology analysis found high concordance in repertoire features between bulk and scBCR-seq within individuals, with the advantage of bulk sequencing providing higher repertoire coverage through higher sampling depth (41). Single-cell BCR sequencing would also allow for detailed heavy and light chain pairing data to be gleaned and longitudinal studies could identify clones which are disease associated. While our study highlights an important aspect of the immunology of MPO-ANCA vasculitis, this is only the beginning of an exciting approach to study additional aspects of autoantigen-specific B cells and the associated BCRs which can also be applied to other autoimmune diseases.

Our data also elicits an interesting discussion on the emergence of disease associated autoantibodies. Our observation that ANCA patients have both disease associated and naturally occurring autoantibodies suggests that epitope spreading may occur from the sites recognized by naturally occurring autoantibodies to portions of the autoantigen not normally recognized by the immune system. Chronic inflammation or specific risk associated HLA alleles may also drive the evolution from naturally occurring to disease associated autoantibodies (42–44). In addition, it is possible that disease specific epitopes could arise via molecular mimicry associated with pathogen infection. In ANCA, as in many autoimmune diseases, studies suggest that similarities between pathogen proteins and endogenous host autoantigens can lead to cross reactive binding of the autoantigen by pathogen-targeting antibodies (45, 46). If those protein similarities occur in regions distinct from the naturally occurring autoantibodies, it is reasonable that they could be a source of disease associated autoantibodies. Alternatively, patients may have other exposures, such as drugs, that induce changes in autoantigens that instigate an immune response. For example, in a study of patients with drug induced ANCA, our group recently described the impact of hydralazine on changes in MPO protein conformation and epitope accessibility leading to autoantibodies to hydralazine modified MPO (47). Similarly, chemical or conformational alterations in autoantigens such as MPO and PR3 may occur during chronic inflammation or other cellular events such as NETosis, leading to release of modified antigens bearing cryptic or neoepitopes (44, 48). Lastly, perturbation of immunoregulatory mechanisms (such as B and T regulatory cells) that normally prevent production of disease associated autoantibodies could allow production of autoantibodies with distinct epitope specificity (49).

Our study highlights differences in naturally occurring MPO ANCAs found in healthy individuals compared to disease-associated autoantibodies through two aspects of the autoantibody: the immunogenomic and epitope binding signatures. This approach needs to be replicated in a larger cohort but one can envision that these findings could be adapted for clinical use in the future. Once validated, identification of ANCA epitopes specific to disease phenotypes could lead to an important diagnostic tool to identify disease state once validated. For example, one could design an ELISA assay to detect disease associated epitopes which distinguish “false positive” or naturally occurring ANCAs. However, ANCA pathology involves far more than the autoantibodies on their own. Additional factors such as glycosylation, complement fixation and even epitope modification may also contribute to eventual immunopathogenesis. Further understanding and potential modification of these various factors will be part of achieving clinical immunological remission rather than simply eradicating all B cells.

## Data Availability

The datasets presented in this study can be found in online repositories. The names of the repository/repositories and accession number(s) can be found below: https://www.ncbi.nlm.nih.gov/geo/ [GSE326295].
