# Supplementary figures and images for: Differentiating naturally occurring and disease associated autoantibodies in MPO-ANCA vasculitis through immunogenomic and epitope comparisons
Source: Front Med (Lausanne). 2026 Jun 2;13:1784607. doi: 10.3389/fmed.2026.1784607 (PMC13270024; doi:10.3389/fmed.2026.1784607)

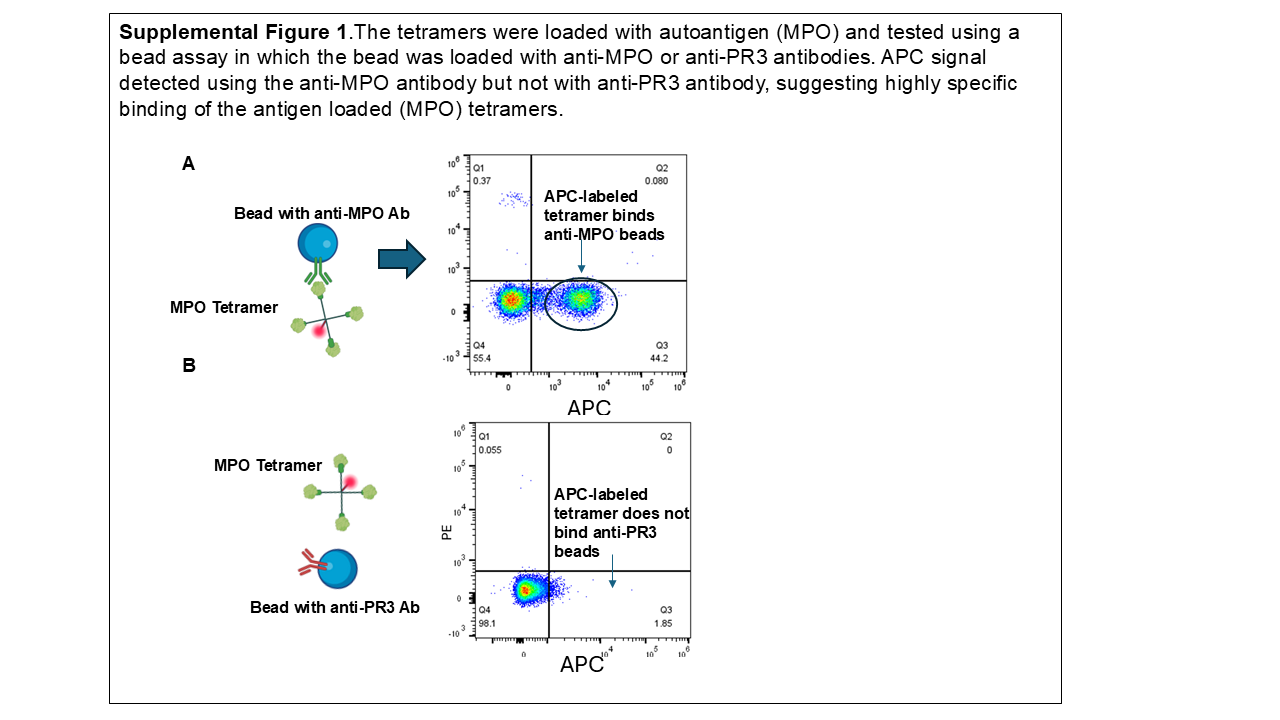

Supplement: Supplementary file 1 [file Image_1.tif]

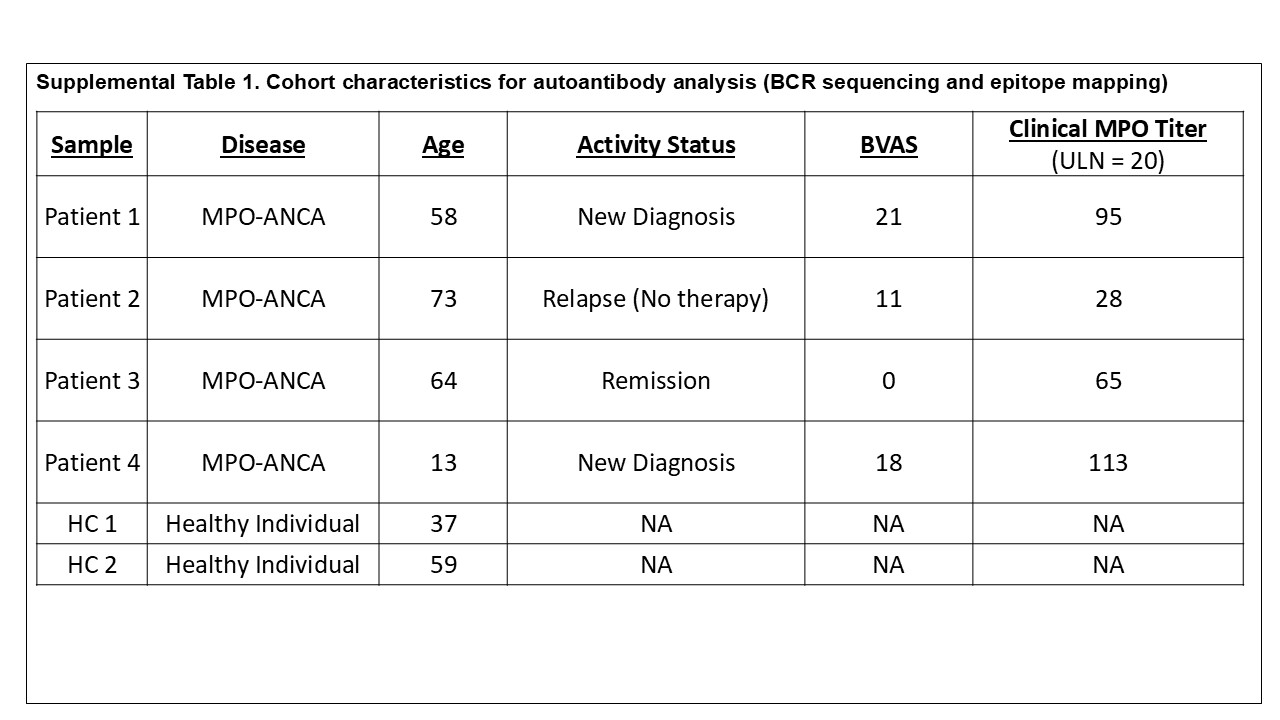

Supplement: Supplementary file 2 [file Table_1.docx]

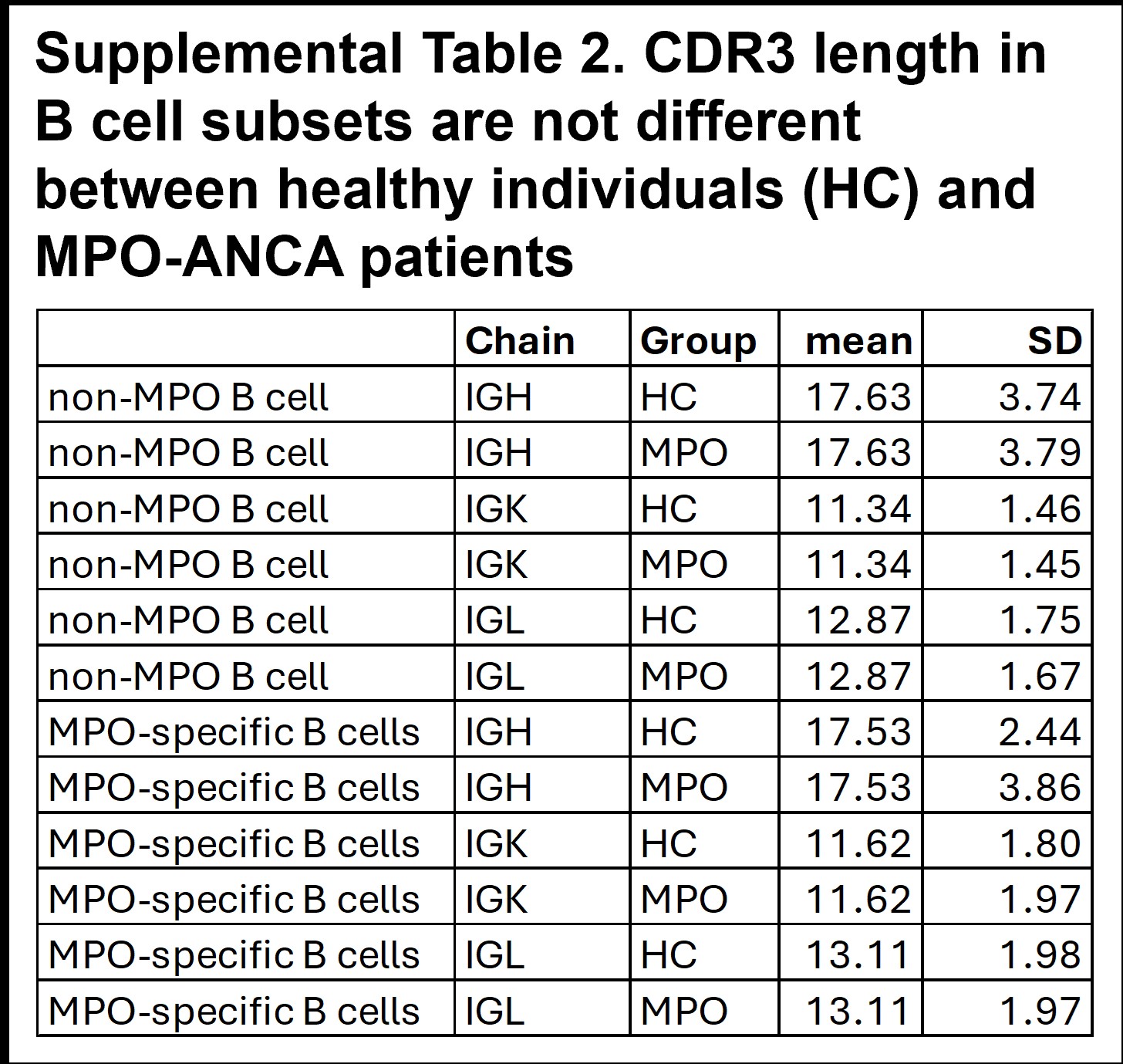

Supplement: Supplementary file 3 [file Table_2.docx]
